# The economic burden of COVID-19 premature mortality in Kuwait

**DOI:** 10.1186/s12889-025-25940-x

**Published:** 2025-12-29

**Authors:** Mohammad Almari, Stephen O’Neill, Zia Sadique

**Affiliations:** 1 Department of Health Services Research & Policy, London School of Hygiene and Tropical Medicine, University of London, London, United Kingdom; 2 Department of Health Policy & Management, College of Public Health, Kuwait University, P.O. Box, Shadadiyah, Kuwait

**Keywords:** Mortality, Years of potential life lost, Human capital approach, Value of statistical life, Friction cost approach

## Abstract

**Background:**

COVID-19 has caused substantial mortality worldwide, with significant economic consequences. In countries with segmented labour markets, such as Kuwait—where most citizens work in the public sector and most non-Kuwaitis occupy high-exposure essential jobs—the economic impact of premature mortality could be considerably high and these losses may differ across population groups. No prior study in the Gulf region has quantified these losses using established valuation methods.

**Methods:**

We conducted a retrospective analysis of all confirmed COVID-19 deaths in Kuwait between 2020 and 2022. Years of Potential Life Lost (YPLL) was calculated to measure the epidemiological burden of premature mortality. The economic cost of premature mortality was estimated from a societal perspective using three approaches: the Value of Statistical Life (VSL), the Human Capital Approach (HCA), and the Friction Cost Approach (FCA). Consumption, wage, and employment parameters were drawn from national 2021 surveys, and all estimates were expressed in 2021 international dollars (PPP$). Sensitivity analyses assessed the influence of key assumptions for each method.

**Results:**

A total of 2,891 COVID-19 deaths occurred during the study period, resulting in approximately 68,000 YPLL, of which 61% were among non-Kuwaitis. Mortality among non-Kuwaiti males was concentrated in working ages, while Kuwaiti deaths occurred primarily in older adults. The total economic burden of premature mortality was estimated at 10.4 billion PPP$ using VSL, 548 million PPP$ using HCA, and 33 million PPP$ using FCA. Kuwaitis accounted for a larger share of VSL and HCA losses, whereas non-Kuwaitis bore the greatest share of YPLL and HCA losses in working ages. Sensitivity analyses showed that VSL results were most affected by discount rate and risk aversion, HCA by age-at-death and wage assumptions, and FCA by vacancy multipliers and friction periods; however, the relative ranking of the methods remained consistent.

**Conclusions:**

Premature COVID-19 deaths in Kuwait generated a significant economic burden, falling most heavily on non-Kuwaiti working-age men. The findings highlight the need for improved occupational protections, stronger support for migrant workers, and targeted preparedness strategies in countries with similar dual labour-market systems.

**Supplementary Information:**

The online version contains supplementary material available at 10.1186/s12889-025-25940-x.

## Background

The COVID-19 pandemic caused substantial global health losses, with premature mortality representing one of the most consequential health and economic impacts [1, 2]. Although mortality was highest among older adults, a considerable share of COVID-19 deaths occurred among individuals in their working years—particularly those aged 30–64—resulting in significant losses of human capital, productivity, and future income [3–5]. Quantifying these losses is critical for informing public-health preparedness, labour policy, and economic resilience planning.

International evidence demonstrates that premature COVID-19 mortality has reduced labour supply, disrupted household income, and imposed long-term economic pressures on health systems and national economies [6, 7]. Studies estimate that early COVID-19 deaths contributed to hundreds of billions in productivity losses globally through diminished earnings, reduced tax revenues, and slower economic growth [3, 8]. A growing body of research shows that indirect costs—particularly those linked to morbidity, premature mortality, and productivity losses—constitute the majority of the pandemic’s economic burden. In China, societal losses of COVID-19 reached US$383 billion in the first quarter of 2020 alone, with 99.8% attributed to productivity disruptions from mobility restrictions and labour-supply shocks [9]. Similar findings have been reported in Iran and Turkey, where mortality-related productivity losses outweighed direct medical expenditures, driven by substantial years of potential life lost (YPLL) in working-age groups [10, 11].

Comparable pattern was reported in high-income countries. A pan-European study estimated €1–3 billion in paid and unpaid productivity losses linked to COVID-19 mortality [12], while U.S. state-level analyses valued death-related losses at US$17 billion in Ohio and US$220 billion in California using Value-of-Statistical-Life (VSL) methods [13]. COVID-related morbidity also produced large temporary productivity losses—equivalent to approximately 9% of U.S. weekly GDP at pandemic peaks [14]. Across settings, premature mortality and productivity losses have disproportionately affected socio-economically vulnerable groups—including racial and ethnic minorities, migrant workers, and frontline occupations [15–21] —demonstrating the unequal distribution of the pandemic’s economic impact. This global evidence highlights the need for country-specific valuation of premature mortality using transparent and rigorous methods that capture both long-term productivity losses and broader societal welfare effects.

Economic valuation of premature mortality is methodologically complex, with three dominant frameworks in the literature: the Value of a Statistical Life (VSL), the Human Capital Approach (HCA), and the Friction Cost Approach (FCA). Each captures a distinct dimension of mortality burden. VSL reflects society’s willingness to pay for mortality-risk reductions, incorporating both market and non-market welfare components [22, 23]. HCA estimates the present value of future earnings lost due to premature death, representing long-term macro-economic productivity loss [24]. FCA focuses on short-term employer-level productivity losses during the period required to replace a deceased worker [25]. These approaches typically yield markedly different estimates; applying multiple methods within a single context; therefore, provides a more comprehensive and policy-relevant assessment.

Kuwait presents a unique and understudied context for examining these frameworks. Of its 4.7 million residents, two-thirds are non-Kuwaiti workers, many employed in private-sector, labour-intensive, and high-exposure occupations [26]. Excess mortality during the pandemic was disproportionately concentrated among non-Kuwaitis, reflecting differences in occupational risk, housing density, and access to health services [27]. Regional reports also point to worse outcomes among South Asian workers compared with Arab populations, highlighting persistent socioeconomic disparities [28, 29]. This dual-population structure—distinguished by divergent wages, living conditions, and social-benefit entitlements—creates two distinct mortality-risk and economic-loss profiles. Despite this, no study in the Gulf region has quantified the economic burden of premature COVID-19 mortality using established valuation methods, nor examined differences between citizens and non-Kuwaiti migrant workers.

To address this gap, the present study provides the first national-level valuation of premature COVID-19 mortality in Kuwait using VSL, HCA, and FCA, integrating epidemiological and economic perspectives. By estimating economic losses across three methodological approaches, and comparing impacts by nationality, gender, and age group, this study offers a comprehensive assessment of the pandemic’s economic burden of premature mortality and generates evidence relevant for public-health preparedness and labour-market policy in countries with dual-population labour structures in the Gulf region.

## Methods

### Study design and perspective

We conducted a retrospective, population-level analysis of all confirmed COVID-19 deaths in Kuwait from 2020 to 2022, using official Ministry of Health mortality records. The study adopted a societal perspective, consistent with international guidance for valuing mortality risk [30, 31], and estimated the economic losses associated with premature deaths employing three approaches commonly used in the global literature: VSL, HCA, and FCA. Each approach captures a different dimension of economic loss, allowing policymakers to interpret results from complementary perspectives.

### Data sources

#### Mortality data

Data on all confirmed COVID-19 deaths were obtained from Kuwait’s Ministry of Health National Centre for Health Information (NCHI) reports. Deaths were identified using the International Classification of Diseases, 10th Revision (ICD-10) codes (U07.1) and (U07.2). Each record included age, gender, and nationality (Kuwaiti/non-Kuwaiti), enabling disaggregation across population groups.

#### Economic data

Economic parameters were obtained from the Kuwait Central Statistics Bureau (KCSB), using for the year of 2021 as the unified price base year. Annual consumption data and average household sizes were obtained from the Household Income and Expenditure Survey (HIES 2021). Age- and gender-specific wages and employment rates, separately for Kuwaitis and non-Kuwaitis were obtained from the Labour Force Survey 2021. The World Bank data for 2021 Purchasing Power Parity conversion factor was (1 Kuwaiti Dinar = 5.2 PPP$) used for international comparability [32]. All detailed parameters inputs are provided in the Additional File (see Tables S1–S6).

#### Years of potential life lost (YPLL)

Two definitions of YPLL were used to distinguish between epidemiological and productivity-related burden. Life-expectancy–based YPLL (*YPLL*_*LE*_) quantified the total epidemiological burden across all age groups, using remaining life expectancy at the midpoint of each age category. Working-life YPLL (*YPLL*_*WR*_) measured the loss of productive years up to age 65 and was applied only in the HCA and FCA models, which focus on labour-market impacts. Deaths occurring above age 65 contributed zero *YPLL*_*WR*_. All formulae, age-specific parameters, and the employment-adjusted deaths and *YPLL*_*WR*_ used for HCA and FCA are provided in the Additional File (see Table S1).

### Economic valuation methods

The economic loss attributable to premature COVID-19 mortality was estimated using three complementary approaches.

#### Value of statistical life

We used a utility-based human capital model, adapted from Becker’s lifecycle framework [33] and operationalised following Sweis et al. [34]. Unlike conventional human capital approaches that rely solely on discounted future earnings, Becker conceptualises health and longevity as components of lifetime utility. The resulting formula—adapted for empirical use—defines the VSL as in (Equation 1) below:

1 $$\:{VSL}_{j}=\:\frac{{C}_{j}^{1-{\upgamma\:}}}{\left(1-{\upgamma\:}\right)\mathrm{r}}$$

Where, *r* is the discount rate, γ is the coefficient of the concavity of the utility function, and *(C*_*j*_*)*​ is the full consumption (market consumption + monetised leisure) for each subgroup (*j)*.

Consistent with Sweis et al. [34], the base-case coefficient of longevity was set to (γ = 0.5), with sensitivity analysis considered plausible range of coefficient. The full consumption (*C₁*) reflects both market consumption and the value of leisure time as [C1​=*x*^∗^ + (*h*_*L*_​×*w*)], where, (*x*^∗^) equivalised household consumption obtained from (HIES 2021), (*h*_*L*_*)* is the annual leisure hours (16 h/day × 365), and (*w*) is the annual wage.

Age-specific consumption profiles were reconstructed using income patterns from Euromonitor, applied to HIES quintile data. This created 75 consumptions cohorts (15 age groups × 5 quintiles) by nationality. Full details are provided in (Additional File: Tables S2–S3).

#### The human capital approach

HCA estimates the present value of future earnings lost due to premature death, up to retirement age (65 years). The annual earnings stream is adjusted for employment probability and discounted. The adjusted economic loss for each age group (𝑖) and category (𝑗) – defined by nationality (Kuwaiti, Non-Kuwaiti) and gender – is shown in (Eq. 2):

2 $$HCA_{jj}=\sum\limits_{a=t_{ij}+1}^{65} \frac{Wj\cdot(Eij \cdot Dij)}{(1+r)(a-ti)}$$

Where, *(W*_*j*_) is annual wage for subgroup $\:\left(j\right)$, (*E*_*ij*_) is the employment rate for age group (*i)* and subgroup (*j)*, (*D*_*ij*_) is midpoint age at death, and *r* is the discount rate (3.5%). Wage bounds used for sensitivity testing are shown in Additional File (Table S4).

#### The friction cost approach

The FCA estimates short-term productivity loss during the period required to replace a deceased worker. The traditional friction period used in the literature is 90 days (3 months) which originated in Koopmanschap et al. [25]. Kuwait’s friction period was estimated following Hanly et al. [35], using a vacancy duration model calibrated with Kuwait’s unemployment and vacancy rates. The estimated duration was 72 days (vacancy duration) and additional 20 days for training and recruitment process, which yielded total 92 days total friction period. Derivations and regression components for this estimation are provided in (Additional File).

For vacancy multiplier, which is the chain of vacancies that would be created for replacing of an employee, we used a vacancy multiplier of 2.14, consistent with labour market structures similar to Kuwait’s, following Hanly et al. [36]. The adjusted economic loss for each age group (𝑖) and category (𝑗) – defined by nationality (Kuwaiti, Non-Kuwaiti) and gender – is shown in (Eq. 3):

3 $$FCA_{ij}=\sum W_{j\:daily} \cdot VD \cdot VM \cdot (E_{ij} \cdot D_{ij})$$

Where, (*W*_*j daily*_) is daily wage for subgroup (*j*), (*VD)* is the friction period, (*VM*) is the vacancy multiplier, (*E*_*ij*_) Employment rate for age group (*i*) and subgroup (*j) and (D*_*ij*_) is number of deaths.

### Sensitivity analyses

To test the robustness of each method, we varied key parameters across plausible ranges based on national data and international guidelines. For the VSL, we varied the discount rate (2–5%), the concavity parameter γ (0.3–0.7), household size (± 20%), and age-consumption profiles (including flat and ± 20% gradients). For the HCA, we varied wage levels (± 20%), discount rate (2–5%), and alternative positions for the age-at-death midpoint. For the FCA, we varied the friction period (60–120 days), vacancy multiplier (± 50% of base), and daily wages (± 20%). These analyses examine how sensitive each method is to reasonable changes in inputs without altering the fundamental ranking of methods. Full sensitivity parameters are provided in the Additional File (Table S6). All sensitivity analyses were performed using R Studio (Version 4.4.0).

## Results

### Descriptive mortality in the population

**Fig. 1:**
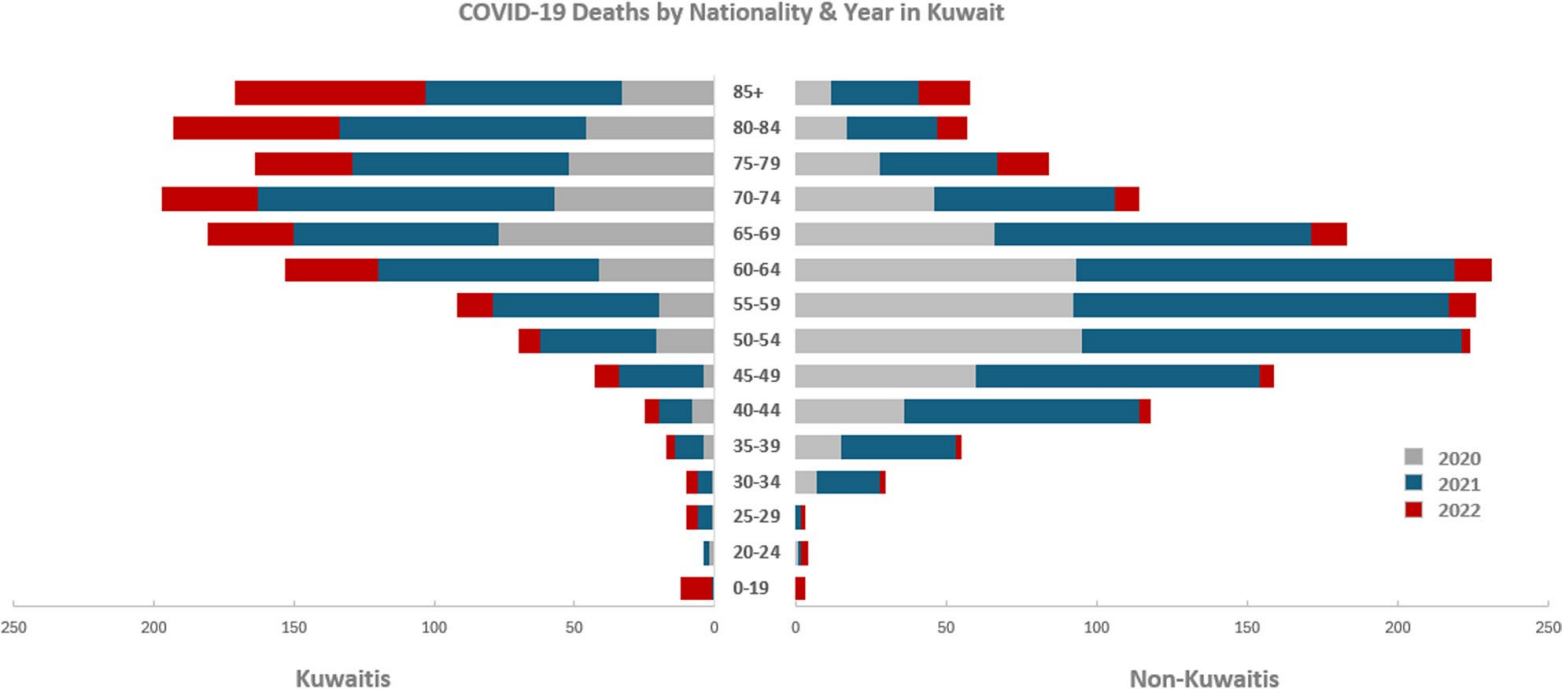
The distribution of COVID-19 deaths by nationality, year & age group (2020 to 2022)

Between 2020 and 2022, a total of 2,891 confirmed COVID-19 deaths occurred in Kuwait (Table 1). Non-Kuwaitis accounted for 53% of all deaths, reflecting their larger share in the working-age population. Most deaths occurred during 2020–2021, with a sharp decline in 2022 following increased vaccination coverage (Fig. 1). Mortality displayed a steep age gradient: 83% of all deaths occurred among adults aged 50 years and above.

**Table 1:** Total deaths and years of potential life lost (*YPLL*_*LE*_) by nationality, gender, & age group (2020–2022)

		Deaths				YPLL^†^							
	% of Total Deaths	Kuwaiti		Non-Kuwaiti		Kuwaiti			Non-Kuwaiti				
Age group		Males	Females	Males	Females	Males	Females			Males		Females	
15–19	0.5%	7	5	0	3	456	348			0		209	
20–24	0.3%	2	2	3	1	120	129			181		65	
25–29	0.4%	5	5	3	0	277	299			166		0	
30–34	1.4%	5	5	17	13	253	274			860		712	
35–39	2.5%	8	9	36	19	366	449			1648		947	
40–44	4.9%	8	17	82	36	328	764			3360		1618	
45–49	7.0%	22	21	116	43	797	841			4202		1723	
50–54	10.2%	40	30	166	58	1263	1057			5243		2044	
55–59	11.0%	47	45	182	44	1274	1373			4933		1342	
60–64	13.3%	70	83	188	43	1597	2150			4290		1114	
65–69	12.6%	93	88	131	52	1749	1889			2463		1116	
70–74	10.8%	80	117	73	41	1203	2030			1098		711	
75–79	8.6%	62	102	50	34	723	1377			583		459	
80–84	8.6%	83	110	30	27	719	1099			260		270	
85+	7.9%	103	68	24	34	629	478			147		239	
Total	100%	635	707	1101	448	11,755	14,557			29,434		12,569	
Mean YPLL	24^‡^					19		21			27		28

^**†**^ The sum of YPLL for each age group as function of subtracted from life expectancy for Kuwaiti nationals and multiplied by number of deaths

^**‡**^ Overall YPLL mean

Patterns of mortality differed markedly by nationality and gender. Non-Kuwaiti males experienced disproportionately high mortality in the 30–64 age group, while Kuwaiti deaths were concentrated primarily in older ages (60+). Deaths in the 0–29 age group were minimal across all subgroups. These patterns reflect Kuwait’s segmented labour market, where non-Kuwaiti males dominate high-exposure, essential occupations (Additional File Figure S1). 

**Fig. 2:**
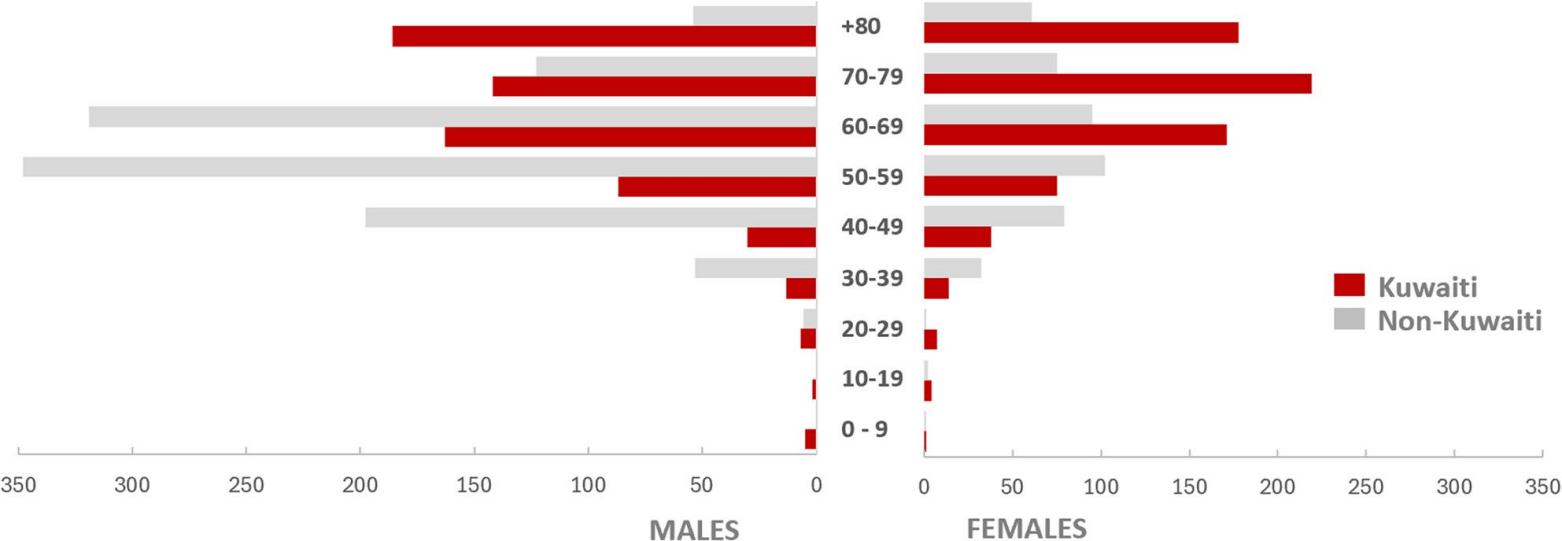
The distribution of COVID-19 deaths by nationality & gender (2020 to 2022

The epidemiological burden captured in *YPLL*_*LE*_ was approximately 68,000 years, of which 61% was among non-Kuwaitis. The crude mean *YPLL*_*LE*_ was 24 years, highest among non-Kuwaiti females (28 years) and non-Kuwaiti males (27 years) (Table 1). Males accounted for 60% of deaths but 68% of *YPLL*_*LE*_, reflecting their higher representation in working-age deaths (Fig. 2).

### The value of statistical life

Using the VSL framework, the total economic loss from premature COVID-19 mortality was estimated at 10.4 billion PPP$, the highest among the three methods (Table 2). Kuwaiti deaths accounted for 57% (5.9 billion PPP$), and non-Kuwaitis 43% (4.4 billion PPP$). The average VSL per death was 4.4 million PPP$ for Kuwaitis and 2.9 million PPP$ for non-Kuwaitis, reflecting differences in consumption levels, wage structures, and household size.

**Table 2:** Economic burden of COVID-19 by approach, nationality and age group (PPP$ Millions)

	VSL		HCA		FCA	
Age group	Kuwaiti	Non-Kuwaiti	Kuwaiti	Non-Kuwaiti	Kuwaiti	Non-Kuwaiti
15–19^†^	53.3	8.7	No cost below 19		No cost below 19	
20–24	17.8	11.6	6.7	1.9	0.2	0.05
25–29	44.4	8.7	19.8	1.2	0.5	0.03
30–34	44.4	87.0	18.5	13.2	0.5	0.3
35–39	75.5	159.5	28.3	22.1	0.8	0.6
40–44	111.1	342.1	34.8	40.5	1.2	1.4
45–49	191.1	461.0	52.7	45.9	2.1	1.8
50–54	311.1	649.5	68.4	49.8	3.5	2.6
55–59	408.8	655.2	59.7	32.3	4.6	2.5
60–64	679.9	669.7	39.6	13.4	7.5	2.5
65–69 ^†^	804.3	530.6	No cost above 65		No cost above 65	
70–74	875.4	330.5				
75–79	728.8	243.5				
80–84	857.6	165.3				
85+	759.9	168.2				
Burden (%)	5,963.5 (57)	4,491.1 (43)	328.4 (60)	220.4 (40)	21 (64)	12 (36)
Total	10,454.6		548.8		33.1	
Per Death	4.4	2.9	1.6	0.47	0.051	0.012

^**†**^ HCA/FCA exclude costs for < 20 & > 65 years

Among Kuwaitis, the VSL burden was greatest in older age groups, particularly 70–74 years (875 million PPP$), where high mortality counts outweighed shorter remaining life expectancy. Among non-Kuwaitis, the economic burden was concentrated in working-age groups, with the highest values in 60–64 (669 million PPP$), 55–59 (655 million PPP$), and 50–54 (649 million PPP$). This pattern reflects the intersection of high exposure in labour-intensive jobs, larger household sizes, and substantial YPLL in these age groups.

### The human capital approach

Total economic loss under the HCA was 548 million PPP$, representing discounted lifetime earnings lost due to premature mortality. Kuwaiti deaths contributed 60% (328 million PPP$) and non-Kuwaitis 40% (220 million PPP$) of the total burden. Average productivity loss per death was 1.6 million PPP$ for Kuwaitis and 0.47 million PPP$ for non-Kuwaitis, reflecting wage differentials between the groups. Among Kuwaitis, the 50–54 and 55–59 age groups together accounted for nearly 40% of total HCA losses, with a peak of 68 million PPP$ in the 50–54 group. Among non-Kuwaitis, losses were more evenly distributed across working ages, with the highest values in 50–54 (49 million PPP$), followed by 55–59 (46 million PPP$) and 45–49 (43 million PPP$).

### Friction cost approach

Using a 92-day friction period, the FCA estimated total economic losses at 33 million PPP$, the lowest among all approaches. Kuwaiti deaths accounted 64% (21 million PPP$), and non-Kuwaitis for 36% (12 million PPP$). Losses were concentrated in ages 50–64, aligning with the ages most relevant for short-term labour market disruptions. For Kuwaitis, the highest burden occurred in 60–64 (7.5 million PPP$), while for non-Kuwaitis it peaked around 50–54 and 60–64 (each approximately 2.6 million PPP$).

**Fig. 3:**
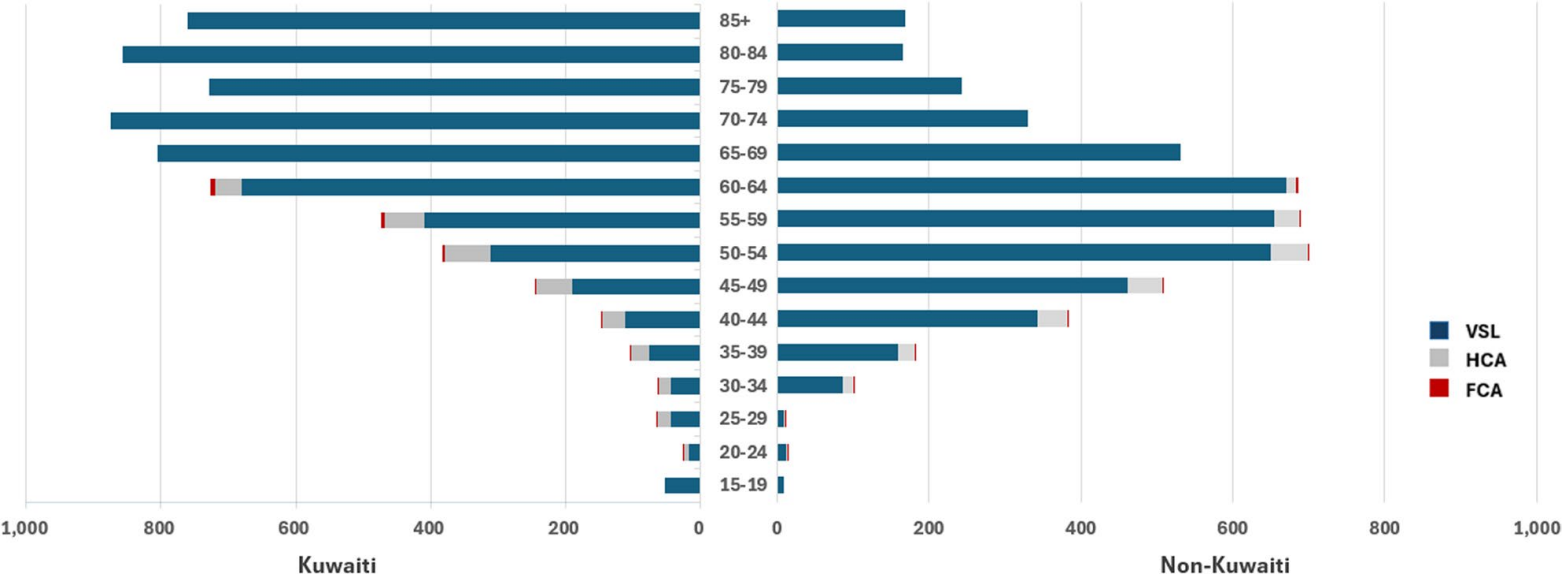
Total costs across three methods by age group & nationality in millions (PPP$)

The scale of estimates differed substantially across methods: VSL estimate was approximately 19 times higher than HCA, and 316 times greater than FCA. A comparison of three approaches by age group and nationality, shows that VSL dominates across all categories, with HCA and FCA providing substantially lower estimates (Fig. 3).

### Sensitivity analysis

**Fig. 4:**
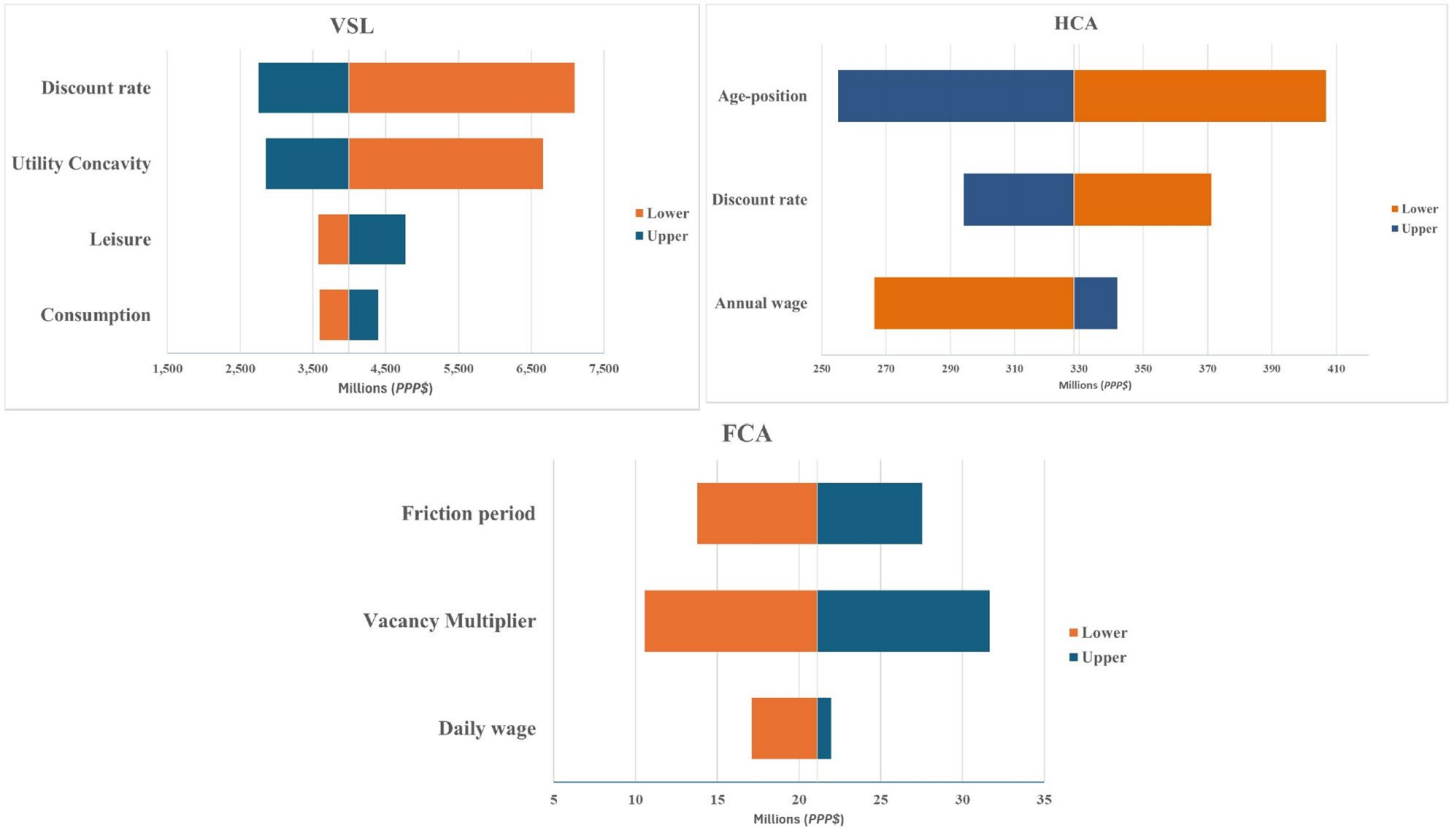
Sensitivity analysis Tornado plots for VSL, HCA, and FCA (PPP$ Millions)

A Tornado plot in (Fig. 4) summarises how changes in key parameters affected each economic valuation. For the VSL, the total burden (base-case: 4 billion PPP$ in the sensitivity subset) was most sensitive to discount rate and concavity factor (γ). A lower discount rate (2%) increased estimates to about 7 billion PPP$, while a higher rate (5%) reduced them to 3 billion PPP$. Varying γ from 0.3 to 0.7 produced a similar range (3.2–6.9 billion PPP$). Leisure and consumption assumptions showed smaller impacts.

For the HCA, the base-case estimate (328 million PPP$) was most affected by age-at-death positioning, increasing to 410 million PPP$ when the midpoint age was reduced, and decreasing to 255 million PPP$ when increased. Wage variations produced a narrower range (270–344 million PPP$), while the discount rate had only modest effects. For the FCA, the total burden (21 million PPP$) was driven mainly by the vacancy multiplier, with estimates ranging from 11 to 32 million PPP$. The friction period produced values between 15 and 27 million PPP$. Wage variation had minimal influence. Overall, VSL estimates were most sensitive to economic preference parameters, HCA to assumptions about remaining working life and wages, and FCA to labour-market frictions. Despite variation, the relative ordering of methods remained unchanged, demonstrating robustness of the main conclusions.

## Discussion

This study provides the first national-level economic valuation of premature COVID-19 mortality in Kuwait, integrating three complementary approaches—VSL, HCA, and FCA—to capture different dimensions of economic loss. The findings show that premature mortality imposed a substantial economic burden, with a total valuation of 10.4 billion PPP$ using VSL approach, 548 million PPP$ using HCA, and 33 million PPP$ using FCA. The wide variation across methods reflects fundamental conceptual differences: VSL captures societal willingness to pay to reduce mortality risk [37]; HCA reflects long-term productivity loss; and FCA reflects short-term employer-level labour-market disruptions [25]. The consistency in the relative ranking of estimates across methods, despite variability in absolute values, underscores the robustness of the results and the importance of applying multiple valuation frameworks when informing policy.

Our findings align with international studies showing that premature mortality represents a major component of COVID-19’s broader economic burden. In China, an early national analysis reported that 99.8% of societal COVID-19 costs stemmed from productivity losses, far exceeding direct medical costs [9]. Similarly, studies from Iran and Turkey found that productivity losses linked to premature deaths and working-age morbidity constituted the majority of economic burden, driven by substantial YLL and YPLL in economically active age groups [11, 38–40]. These observations reinforce our finding that mortality among non-Kuwaiti working-age males—who accounted for a disproportionate share of YPLL—translates into significant economic losses.

In high-income settings, the magnitude of VSL- and HCA-based losses is also comparable. A pan-European study estimated that premature COVID-19 mortality produced €1–3 billion in paid and unpaid productivity losses during the early pandemic period [12]. In the United States, state-level analyses valued COVID-19 mortality at US$17.4 billion in Ohio and US$220 billion in California using VSL approaches [13, 15]. Although Kuwait’s population is significantly smaller, the scale of its VSL burden reflects both substantial mortality in high-income households and the high value placed on risk reduction in high income country contexts. Furthermore, United States data indicate that COVID-related morbidity and quarantine contributed to large temporary losses, with weekly productivity reductions equivalent to 9% of national GDP in some analyses [14]. Our HCA findings echo this trend: while absolute losses are lower in Kuwait due to smaller population size, the concentration of losses in ages 50–64 mirrors patterns observed in Europe and North America.

Notably, international evidence also highlights significant socioeconomic and demographic disparities. In California, the greatest YPLL and VSL losses were concentrated among Latino adults aged 50–64, a group overrepresented in frontline essential jobs [15]. This parallels our findings, where non-Kuwaiti males—who dominate high-exposure occupations and often live in dense, multi-occupancy housing—experienced the highest premature mortality burden. These parallels underscore that structural labour-market inequalities, rather than nationality or ethnicity per se, shape who bears the brunt of economic losses.

Kuwait’s labour-market structure amplifies the economic impact of premature mortality. Non-Kuwaiti males, representing the bulk of the private-sector, manual, and service workforce, experienced both higher mortality in working ages and greater YPLL per death as found elsewhere [41–43]. This has dual implications: a macro-economic impact via long-term productivity loss (HCA) and a micro-economic impact via employer-level disruptions (FCA). For citizens, mortality was concentrated in older ages, resulting in high VSL losses but relatively lower HCA and FCA losses due to lower remaining working years and higher public-sector employment protection.

The FCA estimates, although smaller in magnitude, are important for understanding short-term operational challenges in essential industries such as construction, logistics, and service delivery. Vacancy multipliers and friction periods—major drivers of FCA variability—reflect labour-market tightness and replacement difficulty. In Kuwait, reliance on expatriate labour means that disruptions in recruitment, mobility, or contract renewal can compound the economic impact of premature mortality beyond what is captured in HCA estimates. Additionally, this study complements prior work on the direct medical cost of COVID-19 hospitalisations in Kuwait, which showed high resource use and considerable strain on hospital capacity [44–46]. When combined, these findings reveal a fuller picture: direct medical costs represent only one component, while mortality-related productivity and welfare losses constitute a far larger proportion of the societal burden, mirroring international evidence.

Using three valuation methods strengthens interpretability and policy relevance. Sensitivity analyses demonstrated that the VSL estimates were most influenced by economic preference parameters (discount rate and γ), while HCA was driven primarily by age-at-death and wage assumptions, and FCA by labour-market frictions, particularly the vacancy multiplier. These patterns are consistent with findings from Europe, the United States, and Turkey, where discount rates and remaining working life were critical determinants of mortality valuation outcomes. The consistency of relative rankings (VSL > HCA > FCA) across all sensitivity scenarios enhances confidence that the main conclusions are robust and not dependent on narrow modelling choices. Moreover, cross-referencing consumption, wage, and employment data with national 2021 statistics ensures comparability with international studies that use PPP-adjusted or national price-year inputs [47]. The 17-fold difference between HCA and FCA estimates echoes international findings, where FCA regularly yields lower productivity costs due to its limited time horizon [48].

A major strength of this study is the use of complete national mortality data over a three-year period, allowing stratification by nationality, gender, and age. The integration of VSL, HCA, and FCA within a single national context—particularly in a country with a dual-population labour structure—is innovative and enhances relevance for policymakers. The use of detailed consumption and wage inputs from national surveys, and the inclusion of comprehensive sensitivity analyses, further strengthens methodological transparency. Several limitations warrant consideration. First, the analysis focuses on mortality-related losses only and does not incorporate morbidity-related productivity losses, such as absenteeism due to illness, quarantine, or long COVID. Evidence from international literature suggests that these costs can be substantial and may exceed mortality-related costs in some contexts [3, 5, 7, 49–51]. Second, FCA estimates represent only short-term employer-level disruptions and may underestimate wider economic effects of labour shortages, recruitment delays, or disruptions to essential services [52]. Third, while VSL captures societal valuations of mortality risk reduction, it reflects preferences and consumption patterns specific to high-income settings and may not fully generalise across subpopulations [53].

The findings have important implications for Kuwait and the wider Gulf region. The substantial economic losses associated with premature mortality—particularly among non-Kuwaiti working-age males—underscore the need for targeted occupational health policies, improved housing and living conditions for non-Kuwaiti workers, and strengthened social protection mechanisms. From a public-health perspective, these results support investing in early detection, vaccination, and outbreak preparedness, especially for high-risk occupational groups. From a labour-market perspective, FCA and HCA findings highlight the importance of reducing friction periods, enhancing workforce flexibility, and ensuring continuity in essential sectors during health crises.

## Conclusion

Premature COVID-19 mortality imposed a substantial economic burden on Kuwait, with losses disproportionately borne by non-Kuwaiti working-age males. Using VSL, HCA, and FCA provides a multidimensional understanding of this burden, reflecting societal welfare, long-term productivity, and short-term labour-market impacts. These findings contribute to global evidence on the economic consequences of COVID-19 and offer a methodological template for mortality valuation in countries with dual labour markets. Strengthening occupational protections, expanding social safety nets, and improving health-system preparedness are essential to mitigate similar economic losses in future pandemics.

## Supplementary Information

Supplementary Material 1.

## Data Availability

Data were provided in the additional file and reproducible and can be requested from the corresponding author.

## Abbreviations

VSL: Value of statistical life
HCA: Human capital approach
FCA: Friction cost approach
NCHI: National centre for health information
MOH: Ministry of health
ICD-10: International classification of diseases, 10th revision
KCSB: Kuwait central statistics bureau
HIES: Household income and expenditure survey
YPLL: Years of potential life lost
PPP: Purchasing power parity
