# Supplementary material for: The economic burden of COVID-19 premature mortality in Kuwait
Source: BMC Public Health. 2025 Dec 29;26:405. doi: 10.1186/s12889-025-25940-x (PMC12859997; doi:10.1186/s12889-025-25940-x)
Supplement: Supplementary file 1 — Supplementary Material 1. [file 12889_2025_25940_MOESM1_ESM.docx]

Title: Economic Valuation of COVID-19 Premature Mortality in Kuwait: A Comparative Analysis of Three Methodological Approaches

# Additional file

## 1. Years of Potential Life Lost

We used two definitions of YPLL for different analytical purposes.

1. YPLL-LE (Life Expectancy–Based YPLL)

Used to quantify the total epidemiological burden across all ages.
For each age group $i$:

*YPLL_LE_ =* ∑ *(LE_i_​−t_i_​) × D_i​_*

*i*

Where, *LE_i_* is life expectancy at midpoint of age group, *t_i_​* is the midpoint age at death, *D_i​_* is deaths in age group $i.$

1. YPLL-WR (Working-Life YPLL up to age 65)

Used only in HCA and FCA models because these methods quantify productivity loss:

*YPLL_WR_ =* ∑ *(65−t_i_​​) × D_i​_*

*ti < 65*

Deaths above age 65 contribute zero *YPLL_WR_*. The employment-adjusted deaths and *YPLL_WR_* used in HCA and FCA cost calculations are presented in (Table S1**)**.

Table S1. Employment adjusted deaths and total Years of Potential Life Lost Working life (*YPLL_WR_*) by age group, gender, & nationality

|  |  | **Deaths** | | | |  | **YPLL** | | | |  |
| --- | --- | --- | --- | --- | --- | --- | --- | --- | --- | --- | --- |
|  |  | **Kuwaiti** | | **Non-Kuwaiti** | |  | **Kuwaiti** | | **Non-Kuwaiti** | |  |
| Age groups | Median age | Male | Female | Male | Female | Total Deaths | Male | Female | Male | Female | Total YPLL |
| 15-19 | 17 | 6 | 2 | 0 | 2 | 10 | 288 | 96 | 0 | 96 | 480 |
| 20-24 | 22 | 2 | 1 | 3 | 1 | 7 | 86 | 43 | 129 | 43 | 301 |
| 25-29 | 27 | 5 | 5 | 3 | 0 | 13 | 190 | 190 | 114 | 0 | 494 |
| 30-34 | 32 | 5 | 5 | 17 | 12 | 39 | 165 | 165 | 561 | 396 | 1287 |
| 35-39 | 37 | 8 | 9 | 36 | 19 | 72 | 224 | 252 | 1008 | 532 | 2016 |
| 40-44 | 42 | 8 | 17 | 81 | 35 | 141 | 184 | 391 | 1863 | 805 | 3243 |
| 45-49 | 47 | 21 | 21 | 115 | 43 | 200 | 378 | 378 | 2070 | 774 | 3600 |
| 50-54 | 52 | 39 | 29 | 164 | 57 | 289 | 507 | 377 | 2132 | 741 | 3757 |
| 55-59 | 57 | 46 | 45 | 180 | 42 | 313 | 368 | 360 | 1440 | 336 | 2504 |
| 60-64 | 62 | 68 | 83 | 186 | 41 | 378 | 204 | 249 | 558 | 123 | 1134 |
| Total |  | 208 | 217 | 785 | 252 | 1462 | 2594 | 2501 | 9875 | 3846 | 18816 |

## 2. The VSL method calculation

Annual consumption and wage data from the HIES 2021 capture household income, expenditure, and consumption levels across Kuwait. The KCSB team categorised households into consumption quintiles (Q1 = lowest to Q5 = highest) based on actual reported consumption (Table S1). These quintiles reflect real spending behaviour across the population and were used in the VSL estimation to account for differences in economic valuation across income groups. COVID-19 mortality data were then calculated based to these quintiles using demographic characteristics—specifically age, gender, and nationality—to estimate the differentiated economic burden of premature deaths across population subgroups.

The consumption quintiles were segregated by nationality (Kuwaiti and non-Kuwaiti) to reflect the differential consumption distributions across age groups and nationality (Table S2). To adjust for individual-level consumption, the household data were converted using an equivalence scale based on the square root of household size. Specifically, a household size of (5) was used for Kuwaitis and (2.5) for non-Kuwaitis, in line with estimates from Euromonitor International (2022) (1). Hence, the consumption adjusted by household-equivalence scale is illustrated in (Equation E1).

Equation E1. $x_{j}^{\text{\textbackslash*}}=\frac{x_{j}}{\sqrt{hhsize_{j}}}$

The consumption data according to age groups is not reported by KCSB, thus, data about average gross income in Kuwait by age group for the year 2021 reported by Euromonitor International 2022 was used to estimate a time profile consumption (1). We estimated the percentage change from the overall average income reported in Euromonitor report and multiplied the quintiles in (Table S1) by the precent change derived from report to arrive at the consumption in that quintile in each age group. This step is necessary to account for the variation across different age groups and as data consumption (*C_1_*​) inputs for (in the manuscript Equation 1). The aim is to report the age group and nationality specific consumption quintiles to estimate the weighted VSL mean by age group, and nationality. The results derived from this calculation for Kuwaiti and Non-Kuwaiti are presented in (Table S2).

Table S2. Household consumption (*C_1_*) data by nationality from HIES 2021 presented in (PPP$)

| Kuwait consumption quintiles | | | | | |
| --- | --- | --- | --- | --- | --- |
|  | Q1 (lowest) | Q2 | Q3 | Q4 | Q5 (highest) |
| Monthly | 2,035 (10,582) | 2,937 (15,272) | 3,943 (20,504) | 4,619 (24,019) | 6,196  (32,219) |
| Annual | 126,984 | 183,269 | 246,043 | 288,226 | 386,630 |
| Per capita | 56,789 | 81,960 | 110,034 | 128,898 | 172,906 |
| Non-Kuwait consumption quintiles | | | | | |
|  | Q1 (lowest) | Q2 | Q3 | Q4 | Q5 (highest) |
| Monthly | 856  (4,451) | 1,224 (6,365) | 1,516 (7,883) | 1,632 (8,486) | 2,180  (11,336) |
| Annual | 53,414 | 76,378 | 94,598 | 101,837 | 136,032 |
| Per capita | 33,782 | 48,305 | 59,829 | 64,407 | 86,034 |

Hence, the lifetime consumption profile for a given quintile, is constructed as follows: Ages 15 to 19: 52%. Ages 20 to 24: 75%. Ages 25 to 29: 88%. Ages 30 to 34: 103%. Ages 35 to 39: 115%. Ages 40 to 44: 121%. Ages 45 to 49: 117%. Ages 50 to 54: 105%. Ages 55 to 59: 92%. Ages 60 to 64: 83%. Ages 65 to 69: 73%. Ages 70 to 74: 67%. Ages 75 to 79: 61%. Ages 80 to 84: 57%. Ages above 80: 54%. This step was done for both nationality and have created 75 cohorts for both, five quintiles for 15 age groups. The results derived from this calculation for Kuwaiti and Non-Kuwaiti are presented in (Table S3).

The HEIS 2021 survey is considered one of the most important statistical operations in the field of household surveys and aims to give a comprehensive picture of the various aspects related to the structure and distribution of income and household expenditure. It is a foundation upon which planners, decision makers, and those interested in studying the development of capacity rely on purchasing, prices and living conditions of the population can improve the economy of the State of Kuwait. This report comes after the completion of field work on November 7, 2021, twelve full months later, as well as the completion of office work, auditing, and data review from December 2, 2021.

Table S3. Household consumption quintiles by age group & nationality in 2021 (PPP$)

|  | **Kuwaiti** | | | | |  | **Non-Kuwaiti** | | | | | |
| --- | --- | --- | --- | --- | --- | --- | --- | --- | --- | --- | --- | --- |
| **Age groups** | **Low** | **Moderate** | **Middle** | **High** | **Top** |  | **Low** | **Moderate** | **Middle** | **High** | **Top** |  |
| **15-19** | 29,533 | 42,624 | 57,224 | 67,034 | 89,921 |  | 17,569 | 25,121 | 31,115 | 33,495 | 44,743 |  |
| **20-24** | 42,453 | 61,270 | 82,256 | 96,358 | 129,257 |  | 25,254 | 36,111 | 44,726 | 48,148 | 64,315 |  |
| **25-29** | 50,226 | 72,489 | 97,318 | 114,003 | 152,925 |  | 29,878 | 42,723 | 52,915 | 56,964 | 76,092 |  |
| **30-34** | 58,245 | 84,061 | 112,854 | 132,203 | 177,339 |  | 34,648 | 49,544 | 61,363 | 66,058 | 88,240 |  |
| **35-39** | 65,163 | 94,046 | 126,259 | 147,905 | 198,402 |  | 38,764 | 55,428 | 68,651 | 73,904 | 98,720 |  |
| **40-44** | 68,634 | 99,056 | 132,986 | 155,785 | 208,972 |  | 40,829 | 58,381 | 72,309 | 77,842 | 103,980 |  |
| **45-49** | 66,726 | 96,301 | 129,287 | 151,453 | 203,161 |  | 39,693 | 56,758 | 70,298 | 75,677 | 101,088 |  |
| **50-54** | 59,495 | 85,865 | 115,276 | 135,040 | 181,144 |  | 35,392 | 50,607 | 62,680 | 67,476 | 90,133 |  |
| **55-59** | 52,308 | 75,493 | 101,351 | 118,727 | 159,263 |  | 31,117 | 44,494 | 55,108 | 59,325 | 79,246 |  |
| **60-64** | 47,370 | 68,366 | 91,784 | 107,519 | 144,228 |  | 28,179 | 40,293 | 49,906 | 53,725 | 71,765 |  |
| **65-69** | 41,491 | 59,882 | 80,393 | 94,176 | 126,329 |  | 24,682 | 35,293 | 43,712 | 47,057 | 62,858 |  |
| **70-74** | 38,049 | 54,913 | 73,723 | 86,362 | 115,847 |  | 22,634 | 32,365 | 40,086 | 43,153 | 57,643 |  |
| **75-79** | 34,641 | 49,996 | 67,121 | 78,628 | 105,473 |  | 20,607 | 29,466 | 36,496 | 39,288 | 52,481 |  |
| **80-84** | 32,370 | 46,717 | 62,719 | 73,472 | 98,557 |  | 19,256 | 27,534 | 34,103 | 36,712 | 49,039 |  |
| **85+** | 30,666 | 44,259 | 59,418 | 69,605 | 93,369 |  | 18,242 | 26,085 | 32,308 | 34,780 | 46,458 |  |

## 3. The Human Capital Approach

The HCA focuses on calculating economic loss due to premature mortality by summing the discounted future earnings of the deceased individuals over their remaining working years [maximum to 65 years] adjusting the employment rate. The economic burden from future earnings loss, based on the HCA (Equation 2), was calculated using annual wage data for Kuwaitis and Non-Kuwaitis from the KCSB (2021) (Table S4). Employment-adjusted deaths and the YPLL by age group and nationality are detailed in Table S4. Assumptions used in these estimates were tested in the sensitivity analysis, including the use of the lowest and highest wage bounds.

Table S4. Cost inputs for annual wage earnings by gender & nationality in 2021 (PPP$)

|  | **Kuwaiti** | | **Non-Kuwaiti** | |
| --- | --- | --- | --- | --- |
| Annual wage | Male | Female | Male | Female |
| Base | 112,258 | 78,125 | 19,282 | 29,390 |
| Lower | 94,786 | 59,779 | 18,470 | 25,646 |
| Upper | 116,813 | 81,370 | 47,986 | 42,931 |

Source: Kuwait Central Statistics Bureau (KCSB), 2021

## 4. The Friction Cost Approach

The friction cost approach (FCA) estimates the productivity costs of death from an employer's perspective until another employee or unemployed person takes over the work of the worker who died. The traditional friction period used in the literature is 90 days (3 months) which originated in Koopmanschap et al. (2). This period is set based on the time by which an organisation can hire, train, and restore its production when replacing a worker. The economic loss in this method is exclusively focused on the paid working population. This study adopts an updated version of the friction cost method that includes a country-specific friction period and a vacancy multiplier, following the approach proposed by Hanly et al. (3), as shown in Equation (3).

Kuwait does not consistently report a national vacancy rate. However, its low unemployment rate and high demand for foreign labour suggest a relatively high vacancy rate. To estimate Kuwait’s vacancy multiplier, we referenced countries with similar labour market characteristics—namely Czechia, Germany, the Netherlands, the United Kingdom, and Norway—which exhibit high vacancy rates and comparable GDP per capita levels. Based on the average vacancy multiplier reported for these countries by Hanly et al. (4), Kuwait’s vacancy multiplier was estimated at (2.14).

To estimate the friction period specific to Kuwait, we applied the regression equation detailed in Hanly et al. (3), which follows the vacancy duration linear regression model initially developed using data from the Netherlands by Erdogan et al. (5). The model estimates vacancy duration based on unemployment and vacancy rates, as follows:

*VD_t_* =*β*_0_ + *β*_1_*U_t_* + *β*_2_*V_t_* + *ε_t_*

Where, *(VD*_t_) is the annual vacancy duration, *(U_t_)* is the unemployment rate, and *(V_t_)* is the vacancy rate. The constant and coefficients derived from the equation were applied to unemployment and vacancy rates from 2008 to 2018 to estimate country-specific vacancy durations using Kuwait’s vacancy multiplier of (2.14) and unemployment rate of (2%), along with the intercept. The intercept from the Equation was (β₀ = 43.03) and the coefficients (*U_t_ =* -0.44) and (*V_t_* = 13.87), the estimated friction period for Kuwait is calculated as follows:

43.03 + ((2.14 ∙ 13.87) + (2 ∙ -0.44) = 72 days

A twenty-day training and recruitment period is added to the friction period, giving a total of 92 days. We used the employment-adjusted deaths per age group in (Table S5). The cost parameters used in calculating the total friction cost are shown in Table S5.

Table S5. Cost inputs for 92 days-wage by gender & nationality in (PPP$)

|  | **Kuwaiti** | | **Non-Kuwaiti** | |
| --- | --- | --- | --- | --- |
|  | Male | Female | Male | Female |
| Base | 28,064 | 19,531 | 4,820 | 7,348 |
| Lower | 23,696 | 14,945 | 4,618 | 6,412 |
| Upper | 29,203 | 20,342 | 11,996 | 10,733 |
| Vacancy multiplier | 2.14 | | | |

## 5. Sensitivity analysis

To assess the robustness of the estimated economic burden of mortality using the VSL, a one-way sensitivity analysis was conducted on four key model parameters: the discount rate, utility function concavity (γ), the leisure fraction, and the level of consumption and wage income (*C₁*). Following Sweis (6), a base case value of (γ=0.5) was used in this study, with sensitivity analysis using a range of (0.3) to (0.7). Each parameter was varied across a plausible range (Table S6), while holding all others at their base case values. The study acknowledges that wages may not reflect non-wage benefits or the productivity of informal or unpaid work - a key limitation of the HCA. to address this, wage highest and lowest bounds are tested in the sensitivity analysis.

The assumptions used in the base FCA estimation is further assessed in the sensitivity analysis using highest and lowest daily wage derived from the KCSB of the 92-days wage, and accounting for (±50%) change in the value of the vacancy multiplier.

Table S6. Sensitivity Analysis Parameters

| **Approach** | **Parameter** | **Base Case** | **Range** |
| --- | --- | --- | --- |
| **VSL** | Utility concavity (γ) | 0.5 | 0.3-0.7 |
|  | Full consumption (C_1_) | Table S3 | ±20% of base |
|  | Leisure share (l_1_) | 0.66 | ±20% of base |
| **HCA** | Annual wage | Table S4 | ±20% of base |
|  | Age position | Median-age | Lower/Upper age bound from age-at death midpoint |
| **FCA** | Friction period | 92 days | 60-120 days |
|  | Vacancy multiplier | 2.14 | ±50% of base |
|  | Daily wage | See Table S5 | ±20% of base |
| **ALL** | Discount rate (r) | 3.5% | 2%-5% |

##
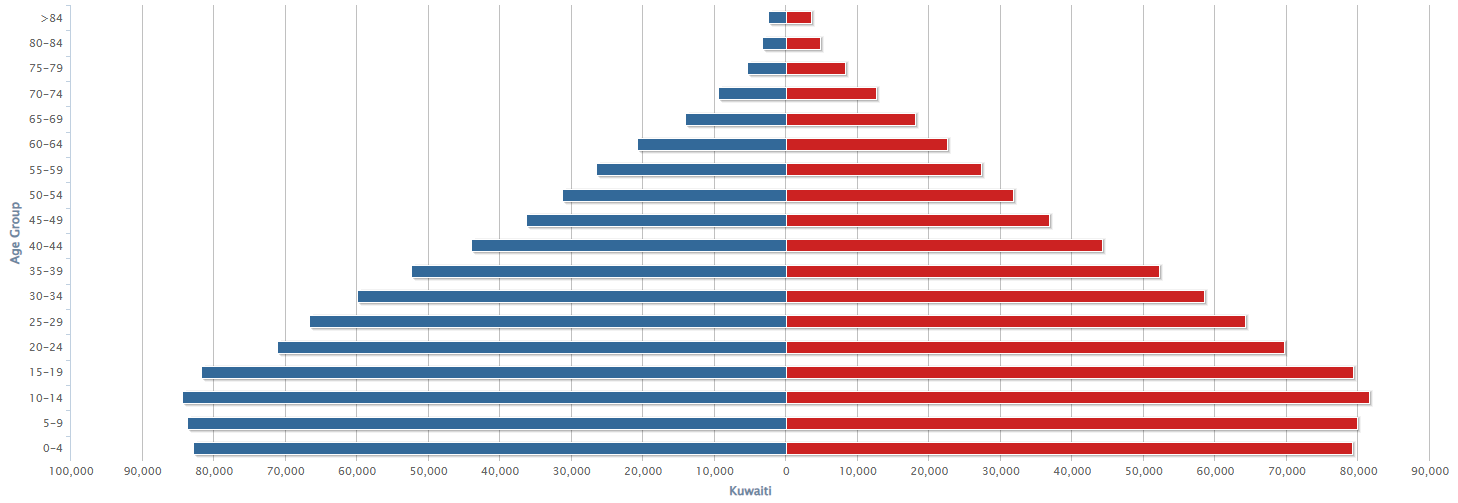
6. Population Distribution


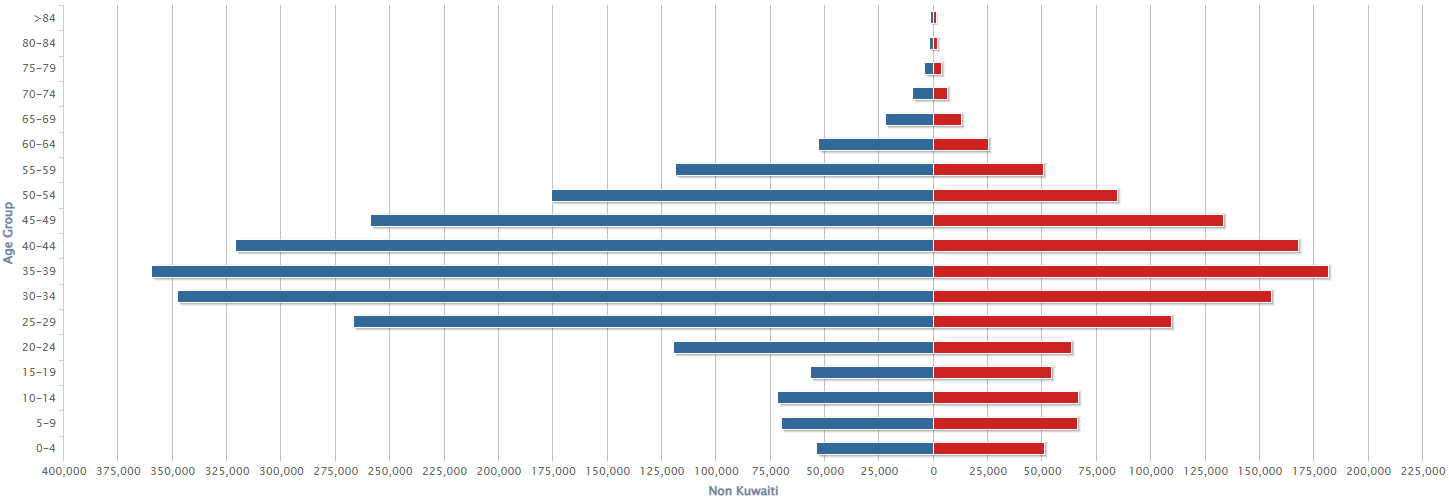


Figure S1. Population distribution for Kuwaiti (A) and non-Kuwait (B) by gender and age groups (June 2025)

## 7. References

1. Government of Canada. Consumer Profile – Kuwait 2023 [Available from: <https://agriculture.canada.ca/en/international-trade/market-intelligence/reports/consumer-profile-kuwait>.

2. Koopmanschap MA, Rutten FF, van Ineveld BM, van Roijen L. The friction cost method for measuring indirect costs of disease. J Health Econ. 1995;14(2):171-89.

3. Paul H, Marta, Alison P, Isabelle S, Linda S. Advances in the methodological approach to friction period estimation: A European perspective. Social Science & Medicine. 2020;264:113289.

4. Hanly P, Ortega-Ortega M, Sharp L. Friction Costs and the Chain of Vacancies Problem: A Novel Vacancy Multiplier Solution. Value in Health. 2021;24(4):548-55.

5. Erdogan E, Koopmanschap M, Bouwmans-Frijters C, Hakkaart L. Productivity costs using the friction cost approach: new evidence using data from several European countries. Technical University report, Department of Health Policy and Management, Erasmus University Rotterdam. 2011.

6. Sweis NJ. Revisiting the value of a statistical life: an international approach during COVID-19. Risk Management. 2022;24(3):259-72.
